# Efficient Delivery of DNA and Morpholinos into Mouse Preimplantation Embryos by Electroporation

**DOI:** 10.1371/journal.pone.0043748

**Published:** 2012-08-21

**Authors:** Hui Peng, Yongyan Wu, Yong Zhang

**Affiliations:** College of Veterinary Medicine, Northwest A&F University, Key Laboratory of Animal Biotechnology, Ministry of Agriculture, Yangling, Shaanxi, People’s Republic of China; Baylor College of Medicine, United States of America

## Abstract

Mouse preimplantation development is characterized by three major transitions and two lineage segregations. Each transition or lineage segregation entails pronounced changes in the pattern of gene expression. Thus, research into the function of genes with obvious changes in expression pattern will shed light on the molecular basis of preimplantation development. We have described a simplified and effective method–electroporation–of introducing plasmid DNA and morpholinos into mouse preimplantation embryos and verified effectiveness of this approach by testing the procedure on the endogenous gene *Oct4*. Before electroporation, the zona pellucida was weakened by the treatment of acid Tyrode’s solution. Then we optimized the parameters such as voltage, pulse duration, number of pulses and repeats, and applied these parameters to subsequent experiments. Compared with the control groups, the number of apoptotic cells and the expression and localization of OCT3/4 or CDX2 was not significantly changed in blastocysts developed from 1-cell embryos, which were electroporated with pIRES2-AcGFP1-Nuc eukaryotic expression vector or mismatched morpholino oligonucleotides. Furthermore, electroporated plasmid DNA and morpholinos targeting the endogenous gene *Oct4* were able to sharply down regulate expression of OCT4 protein and actually cause expected phenotypes in mouse preimplantation embryos. In conclusion, plasmid DNA and morpholinos could be efficient delivered into mouse preimplantation embryos by electroporation and exert their functions, and normal development of preimplantation embryos was not affected.

## Introduction

One essential method to understand the genetic mechanisms controlling embryonic development contains governing gene expression and analyzing the subsequent effects on developmental processes. Introduction of exogenous genes into embryos and targeting of given genes by homologous recombination are conventional and classical techniques to research gene function. Traditionally, the introduction of genes into cells or genomes is dependent on transfection [1], [2], viral infection [3], [4] or microinjection [5], [6], while loss-of-function of genes relies on knock-down or knock-out technologies [7], [8]. However, as far as the stage of preimplantation embryo development in the mouse is concerned, introduction of genes into preimplantation embryos by transfection or viral infection is difficult as embryos are encompassed by zona pellucida. Knock-out technology is not widely used, as it is a time-consuming, laborious and expensive process. To date, exogenous genes and sequences including double-stranded RNA (dsRNA), small interfering RNA (siRNA) and morpholino antisense oligonucleotides for knock-down gene expression have been introduced into mouse zygotes or blastomeres by microinjection [9], [10], [11], [12]. It is not efficient as a method of introducing these molecules into all of the blastomeres of embryos at the 2-cell to morula stage, and only a single embryo can be microinjected at any one time, although this approach is likely to be valuable for following the gain or loss of gene function in a single blastomere.

For years, electroporation has been an effective method for introducing DNA into *Drosophila* [13], insect [14], zebrafish [15], [16], [17], *Xenopus laevis* [18] and chicken embryos [19], [20], [21]. Apart from DNA, antisense morpholinos, bounded to and blocked translation of mRNA, have been successfully delivered into cells or embryos by electroporation [17], [22], [23], [24]. This technique is based on electric pulses generating transient pores in the plasma membrane through which molecules can enter. It is universally accepted that electropermeabilization and electrophoresis are pivotal for successful electroporation; other mechanisms such as passive diffusion and electroendocytosis might also play a role in electroporation.

Electroporation has previously been mainly applied on cells and postimplantation embryos in the mouse [24], [25], [26], [27], [28], [29]. Although some literature involved electroporation that introduced DNA into mouse preimplantation embryos, their protocols injected DNA into the perivitelline space (between the plasma membrane and zona pellucida) of zygotes, and then subjected them to electroporation with very low efficiency [30]. Recently, electroporation was also successfully used to deliver dsRNA and siRNA into mouse preimplantation embryos [31], [32]. However, the electroporation of morpholinos introduced into mouse preimplantation embryos has not been reported. Here we describe a simplified and effective method–electroporation–of introducing DNA and morpholinos into mouse preimplantation embryos and verify effectiveness of this approach by testing the procedure on the endogenous gene *Oct4*. This approach will facilitate the analysis of gene function and extend the knowledge of zygote to blastocyst development in the mouse.

**Figure 1:**
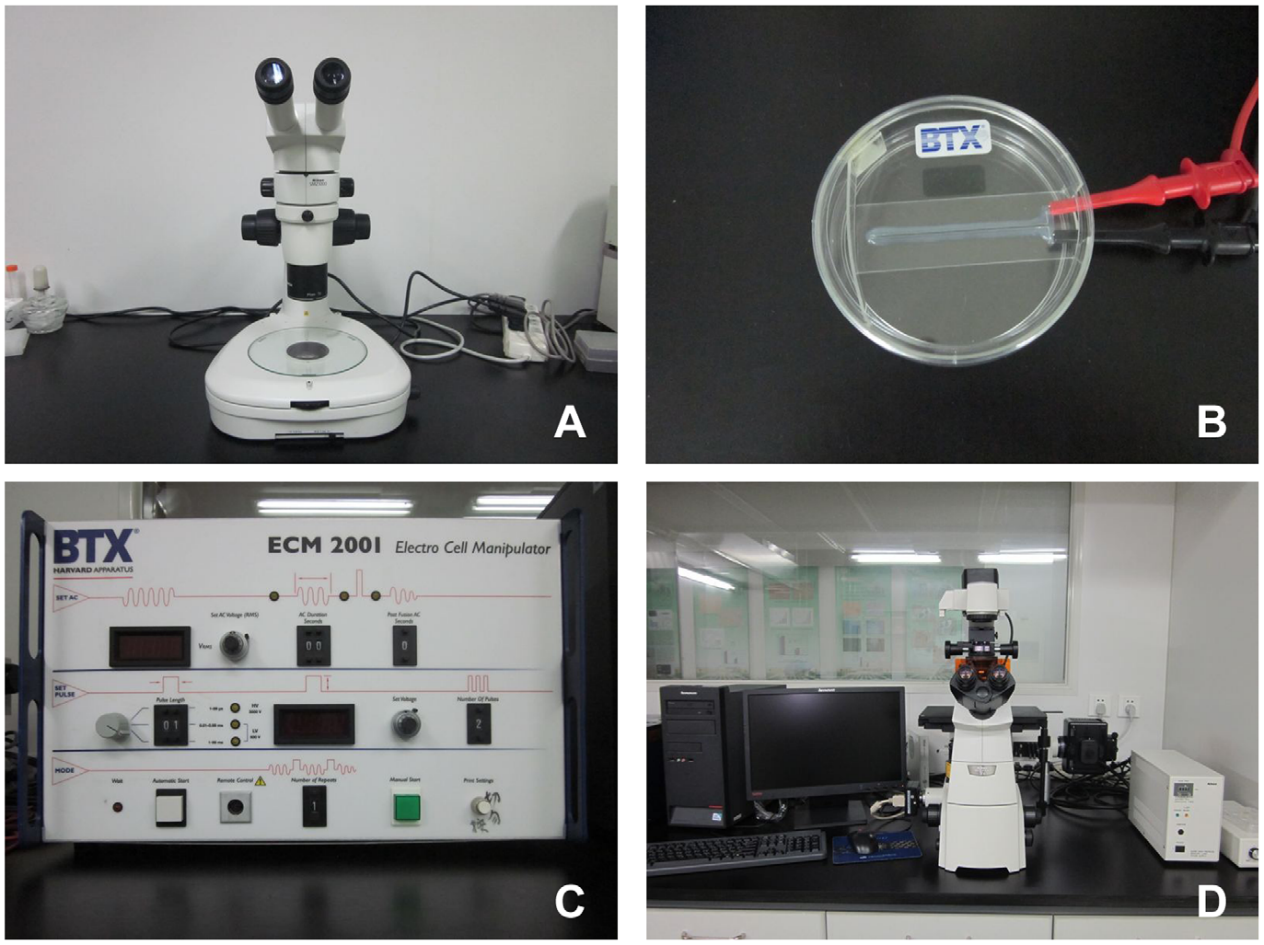
Apparatus for embryo manipulation, electroporation and image capture. (A) A stereoscopic microscope for embryo manipulation. (B) Chamber-type electrodes with 1-mm gap used in this experiment. (C) Electro Cell Manipulator for generating electric pulses. (D) Nikon DS-Ri1 digital camera for image capture.

## Materials and Methods

### Ethics Statement

The entire experimental procedure was approved by the Animal Care Commission of the College of Veterinary Medicine, Northwest A&F University. Adult male and female ICR mice were purchased from the Experimental Animal Center of the Fourth Military Medical University (Xi’an, China). They were maintained on a 14L:10D cycle with free access to food and water in the Laboratory Animal Facility of the College of Veterinary Medicine, Northwest A&F University.

**Figure 2:**
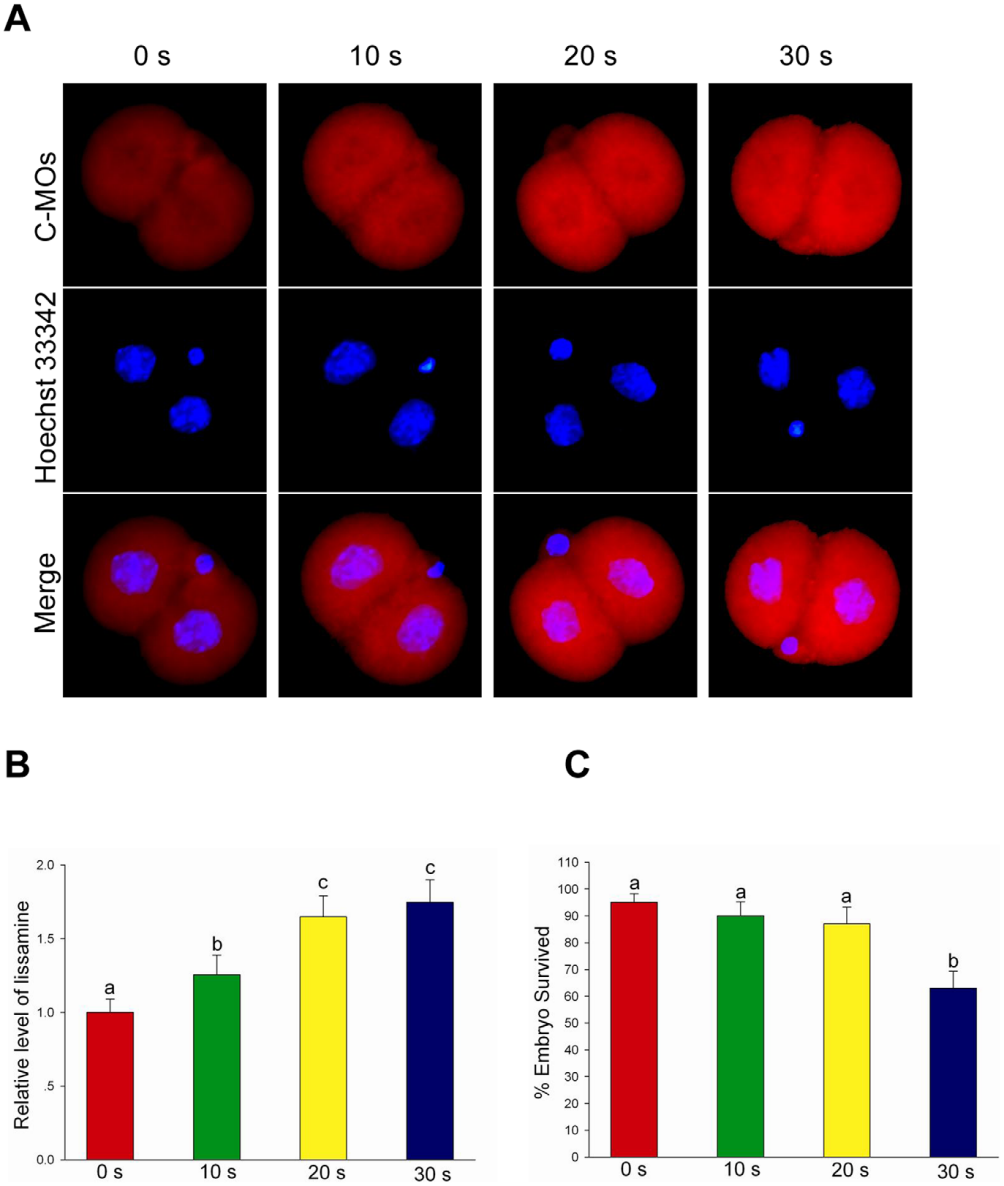
Effect of treatment times of zona pellucida on the electroporation efficiency. (A) Fluorescence level of the standard control morpholinos (C-MOs) in the 2-cell stage embryos when 1-cell embryos were treated with acid Tyrode’s solution, electroporated and cultured for 24 h *in vitro*. Each sample was counterstained with Hoechst 33342 to visualize DNA (blue). Original magnification was ×200. (B) Quantification of the lissamine conjugated morpholinos signal intensities in treated embryos that were incubated in acid Tyrode’s solution for different durations. Labeling intensity was expressed relative to that of the treated 0 s embryos (set as 100%). Values with different superscripts differ significantly (P<0.05). The experiments were replicated 4 times. In each replication, n = 10–15 per group. (C) Embryonic survival rate following electroporation of 1-cell stage embryos incubated in acid Tyrode’s solution for different durations.

### Chemicals

All chemicals and reagents were purchased from Sigma-Aldrich (St. Louis, USA) unless stated otherwise. Sterile plasticware was purchased from Nunclon (Roskilde, Denmark).

### Collection and Culture of Embryos

Zygotes (1-cell embryos) were obtained from 8–10-week-old female mice superovulated with 10 IU of pregnant mare serum gonadotrophin and 10 IU human chorionic gonadotrophin 48 h later and mated with males. Zygotes were released from ampullae of oviducts 20 h after human chorionic gonadotrophin and cumulus cells were removed by hyaluronidase (1 mg/ml) treatment and pipetting in Hepes-buffered KSOM medium [33]. Collection of embryos (2-cell, 4-cell, 8-cell and morula) was performed according to previously described protocols [32]. Embryos were cultured in groups of 30–50 in microdrops (100 µl) of KSOMaa medium [34] supplemented with 4 mg/ml bovine serum albumin (KSOMaa-BSA) under liquid paraffin oil in a humidified atmosphere of 5% CO_2_/95% air at 37°C.

**Figure 3:**
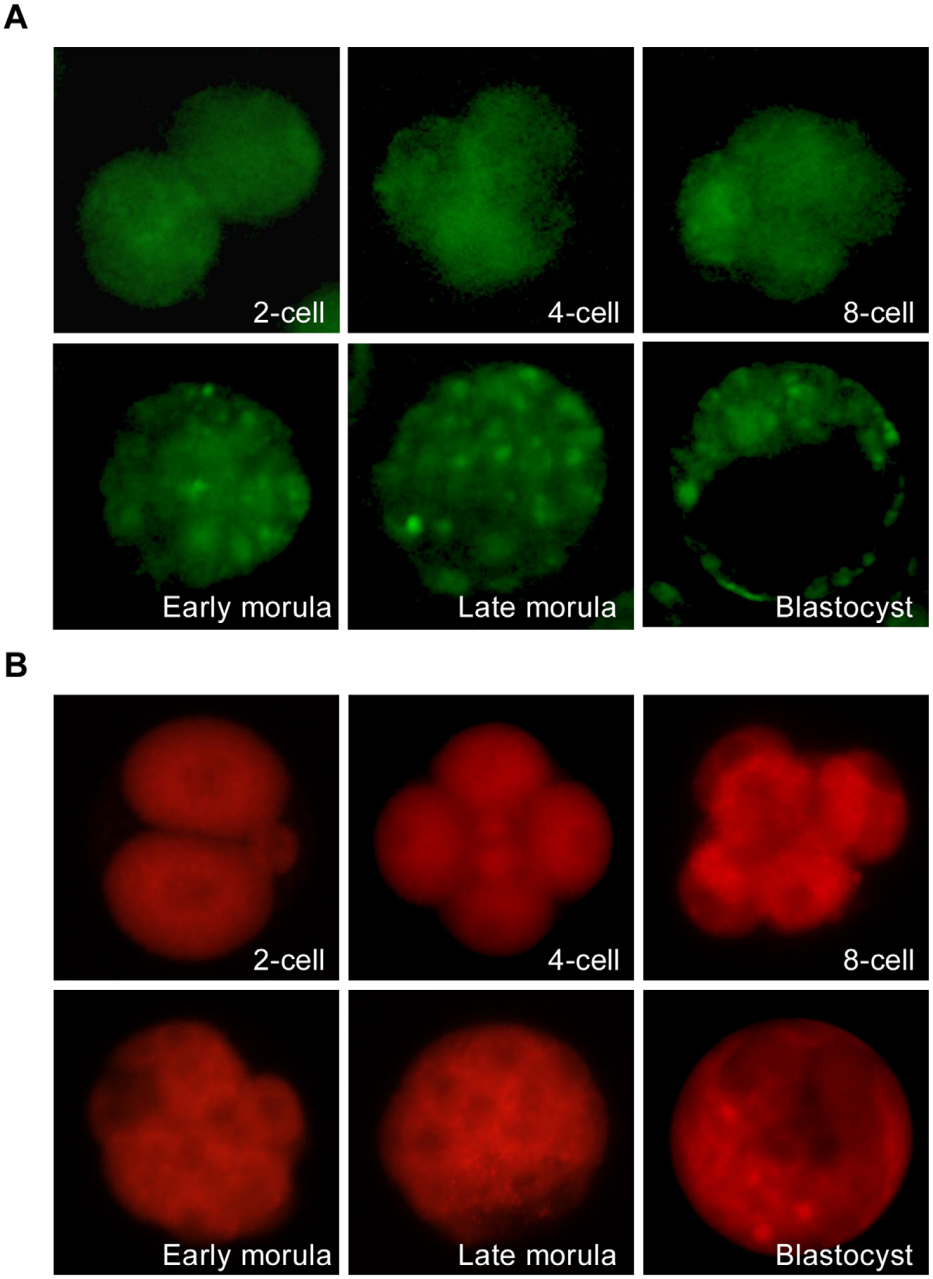
Plasmid DNA and morpholinos were efficiently introduced into mouse preimplantation embryos by electroporation. 1-cell embryos, 2-cell embryos, 4-cell embryos, 8-cell embryos and morulas were electroporated with pIRES2-AcGFP1-Nuc (A) or the lissamine conjugated morpholinos (B) and cultured for 24 h. pIRES2-AcGFP1-Nuc and morpholinos introduce, revealed by green fluorescent protein and lissamine fluorescence, respectively, is efficient. 4-cell embryos and 8-cell embryos were all developed from the electroporated 2-cell embryos. Original magnification was ×200.

### Preparation of DNA and Morpholinos Solution

pIRES2-AcGFP1-Nuc (Clontech, Otsu, Japan) eukaryotic expression vector (pIRES2) was purified by routine methods and finally diluted into Opti-MEM® I (Invitrogen, Carlsbad, CA, USA) medium with a concentration of 40 µg/ml for electroporation. The gene-specific shRNA expression plasmids (Origene, TG511384, Origene Technologies, Inc., Rockville, MD, USA) for specifically knocking down mouse *Oct4* expression (*Oct4*-shRNA) were diluted into Opti-MEM® I medium with the different concentrations (0, 10, 40 and 100 µg/ml). The blank Opti-MEM® I medium was used as electroporation control (EP). An empty vector without shRNA cassette insert (Origene, TR30007) was used as vector control (V-C). The vector containing noneffective 29-mer scrambled shRNA cassette (Origene, TR30013) was used as a negative control (N-C).

**Table 1:** Electroporation conditions and efficiencies for DNA and morpholinos.

Molecule	Stage	Optimal parameters^a^	Survival rate (%)	Electroporation rate for survivors (%)	Numbers of embryos electroporated
DNA	1-cell	30 V; 1 ms; 2×; 1	85	93	112
	2-cell		87	96	108
	4-cell		89	96	97
	8-cell		87	97	121
	Morula		88	94	98
Morpholinos	1-cell	20 V; 1 ms; 2×; 1	91	96	132
	2-cell		90	95	113
	4-cell		91	95	107
	8-cell		92	92	96
	Morula		94	93	105

a The electroporation parameters contain voltage, pulse duration, number of pulses and repeats. Three replicate experiments were performed per embryonic stage.

The morpholino oligonucleotides were purchased from Gene-Tools, LLC (Corvallis, OR, USA). The standard control oligo sequence was 5′–CCTCTTACCTCAGTTACAATTTATA–3′ and modified with lissamine to visualize within the electroporated embryos. To optimize the treatment times with acid Tyrode’s solution for weakening the zona pellucida, this modified morpholinos were diluted in Opti-MEM® I medium with a concentration of 0.2 mM for electroporation. The *Oct4* morpholino oligonucleotides (*Oct4*-MOs) sequence was 5′-AGTCTGAAGCCAGGTGTCCAGCCAT-3′. *Oct4*-MOs were diluted into Opti-MEM® I medium with the different concentrations (0, 0.2, 0.3 and 0.4 mM). The blank Opti-MEM® I medium was used as electroporation control (EP). The standard control oligo was used as a control morpholino (C-MOs). The mismatched oligo sequence was 5′-ACTCTCAAGCCACGTGTGCAGCGAT-3′ and used as a negative control (M-MOs).

**Figure 4:**
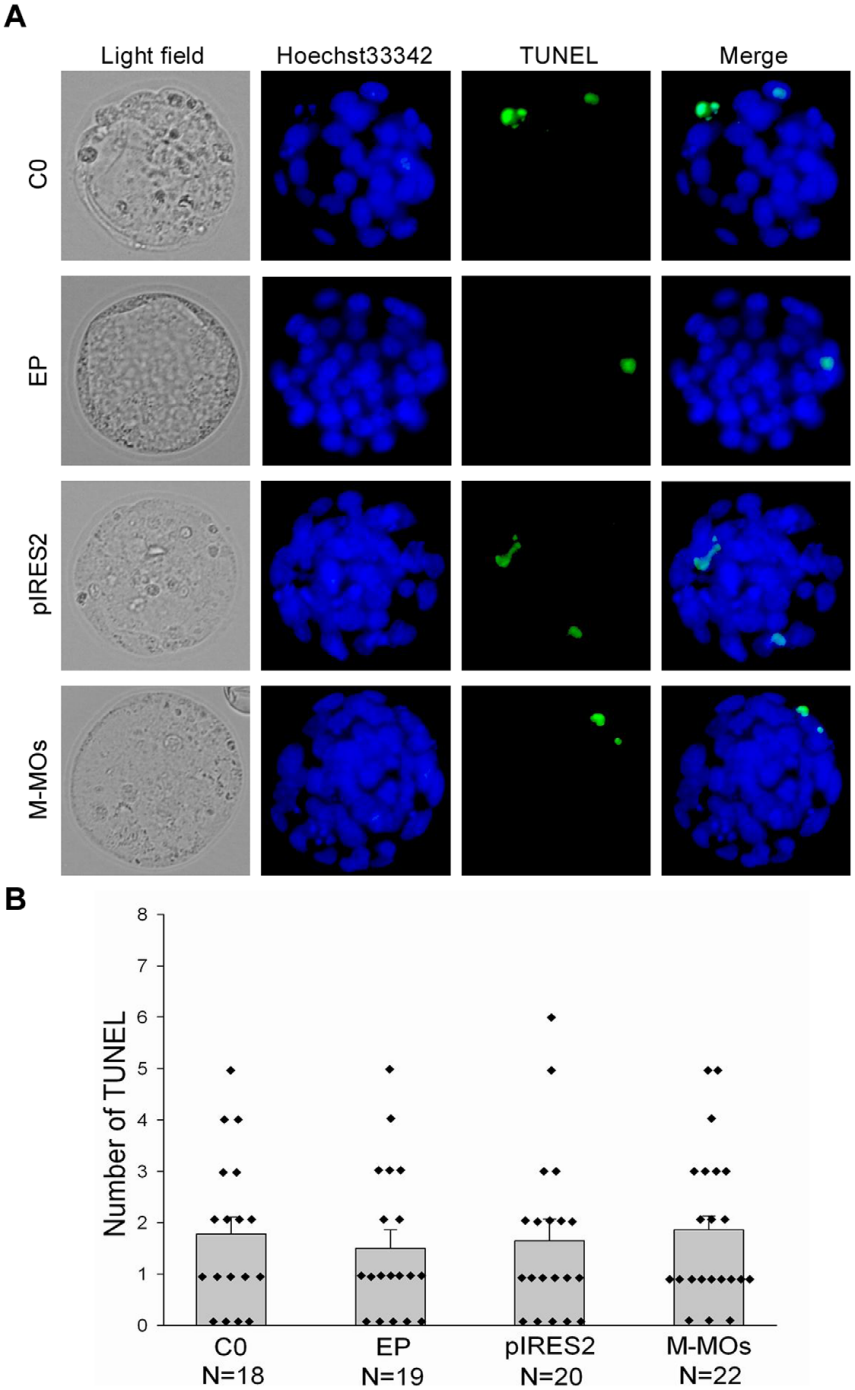
Incidence of apoptosis in blastocysts developed from unelectroporated embryos (C0), EP group, pIRES2-AcGFP1-Nuc (pIRES2) and mismatched morpholino oligonucleotides (M-MOs) electroporation. (A) TUNEL apoptosis assay of blastocysts (green). Each sample was counterstained with Hoechst 33342 to visualize DNA (blue). The original magnification was ×200. (B) Number of apoptotic cells in each blastocyst.

### Apparatus for Electroporation

Embryos were manipulated under a stereoscopic microscope (Nikon, Tokyo, Japan) (Fig. 1A) and then electroporated in a flat electrode chamber (Fig. 1B) supplied by BTX Inc. Square pulses were delivered using a 2001 Electro Cell Manipulator (ECM 2001; BTX, San Diego, CA) (Fig. 1C). Fluorescence was visualized using a Nikon Eclipse Ti-S microscope equipped with a 198 Nikon DS-Ri1 digital camera (Nikon) (Fig. 1D).

**Figure 5:**
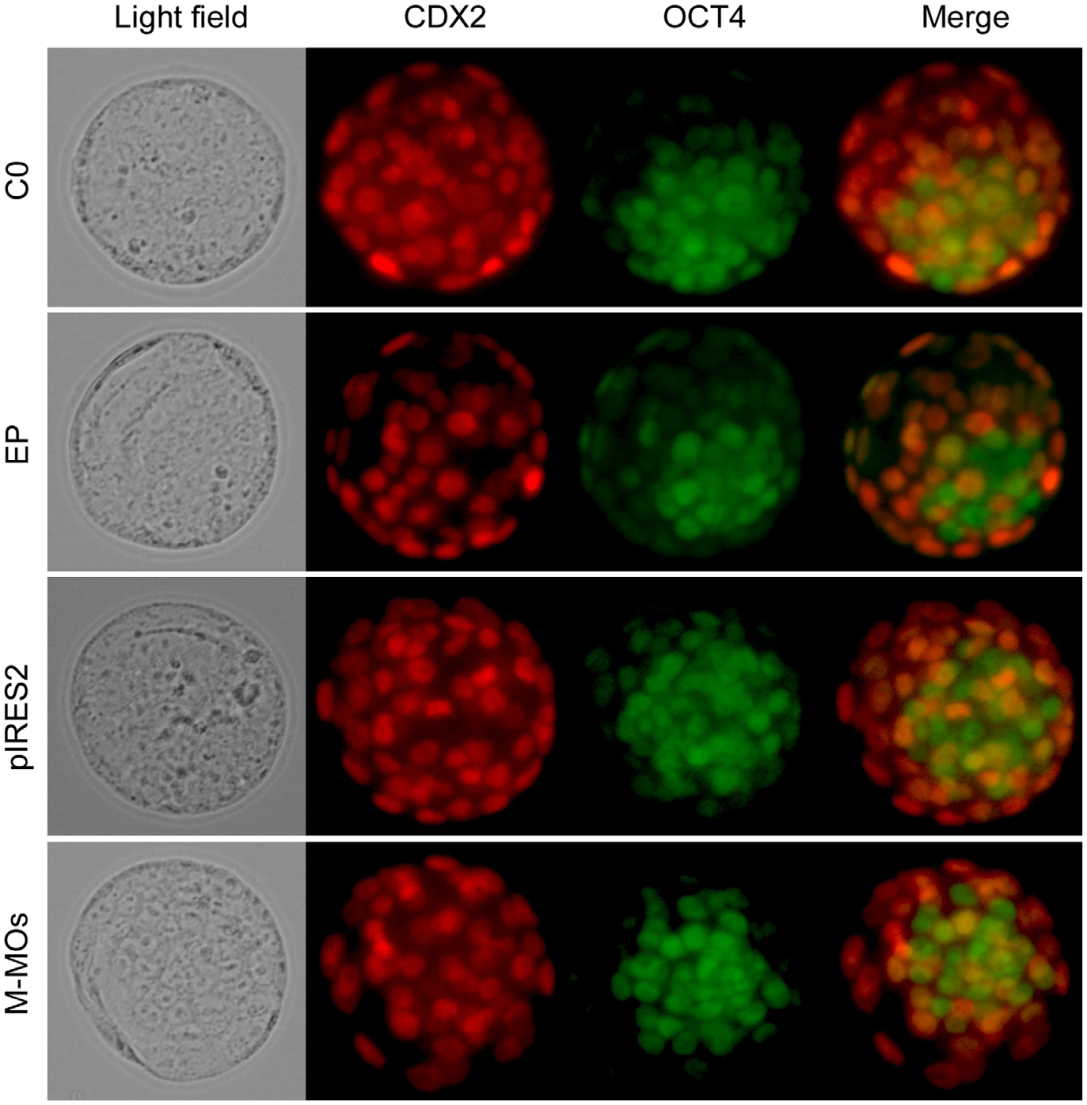
Effects of electroporation on cell differentiation in blastocysts. Immunostaining of CDX2 (red) and OCT4 (green) in blastocysts developed from unelectroporated embryos (C0), EP group, pIRES2-AcGFP1-Nuc (pIRES2) and mismatched morpholino oligonucleotides (M-MOs) electroporation. The original magnification was ×200.

### Optimization of Treatment of Zona Pellucida and Electroporation Parameters

Freshly collected embryos at the different stages were washed in H-KSOM and then incubated in pre-warmed acid Tyrode’s solution for 0, 10, 20 or 30 s to weaken the zona pellucida rather than remove it. Zona-weakened embryos were washed twice in fresh H-KSOM and prepared for electroporation.

**Figure 6:**
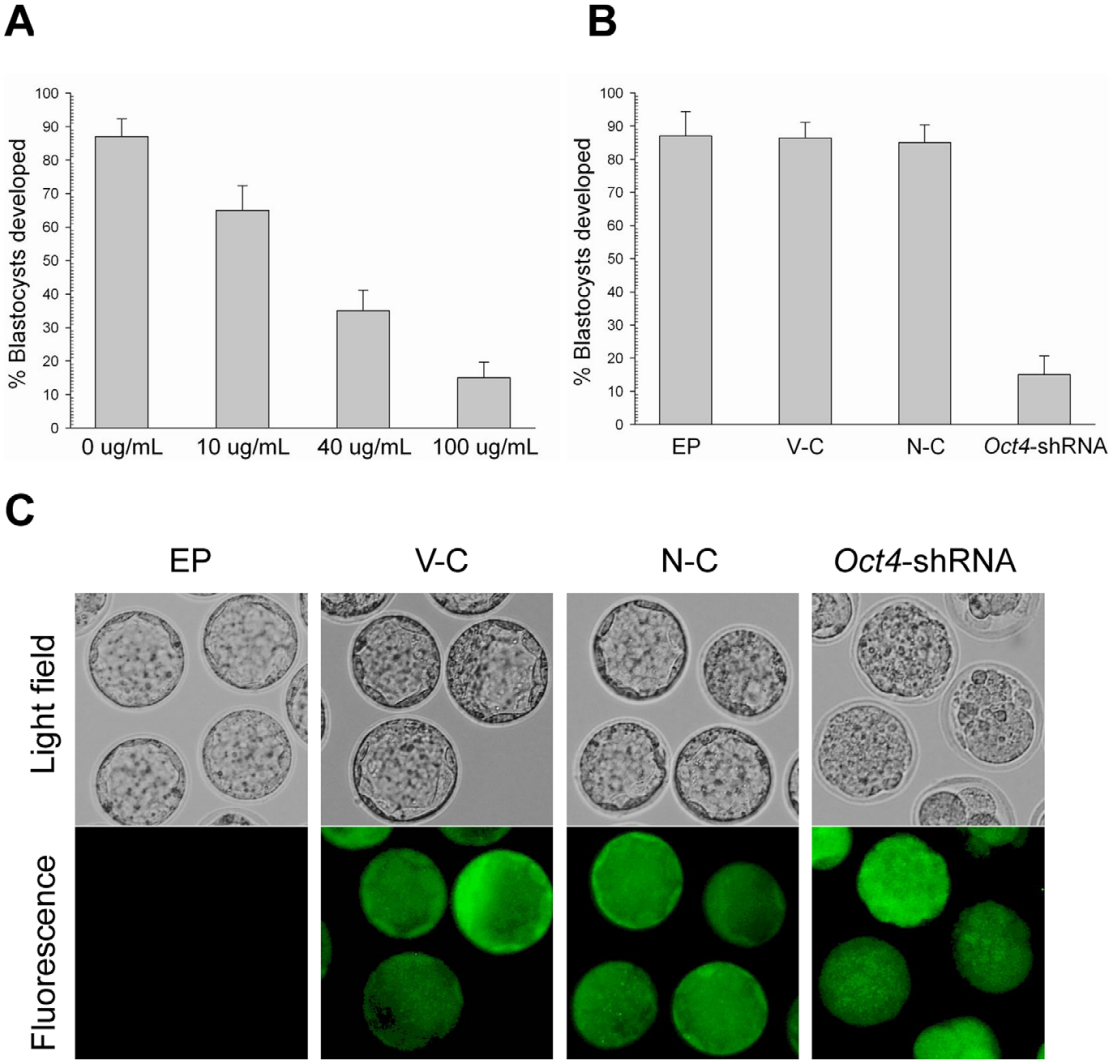
Electroporation of *Oct4*-specific shRNA expression vectors. (A) Blastocyst development following electroporation of zygotes with custom *Oct4*-specific shRNA expression vectors at different concentrations and cultured for 3.5 d. (B) Blastocyst development following electroporation of zygotes with different shRNA expression vectors with a concentration of 100 µg/ml. (C) Morphological appearance of electroporated zygotes obtained from the control and *Oct4*-shRNA groups after being cultured for 3.5 d. Morphology (top) and fluorescence (bottom). The original magnification was ×100.

To optimize the treatment times of zona pellucida with acid Tyrode’s solution, zona-weakened 1-cell embryos were electroporated with modified morpholino oligonucleotides. After culturing for 24 h, embryos were stained with Hoechst 33342 (C1025; Beyotime, Jiangsu, China) for 30 min, and then observed using a Nikon Eclipse Ti-S microscope (Nikon). Images were obtained with the same exposure times and adjustments of the microscope. The intensity of lissamine fluorescence was analyzed using MetaMorph software (Version 6.1; Universal Imaging Corporation) as described previously [35], [36]. To quantify fluorescence intensity, the values of the treated embryos were presented relative to the mean value of untreated embryos.

**Figure 7:**
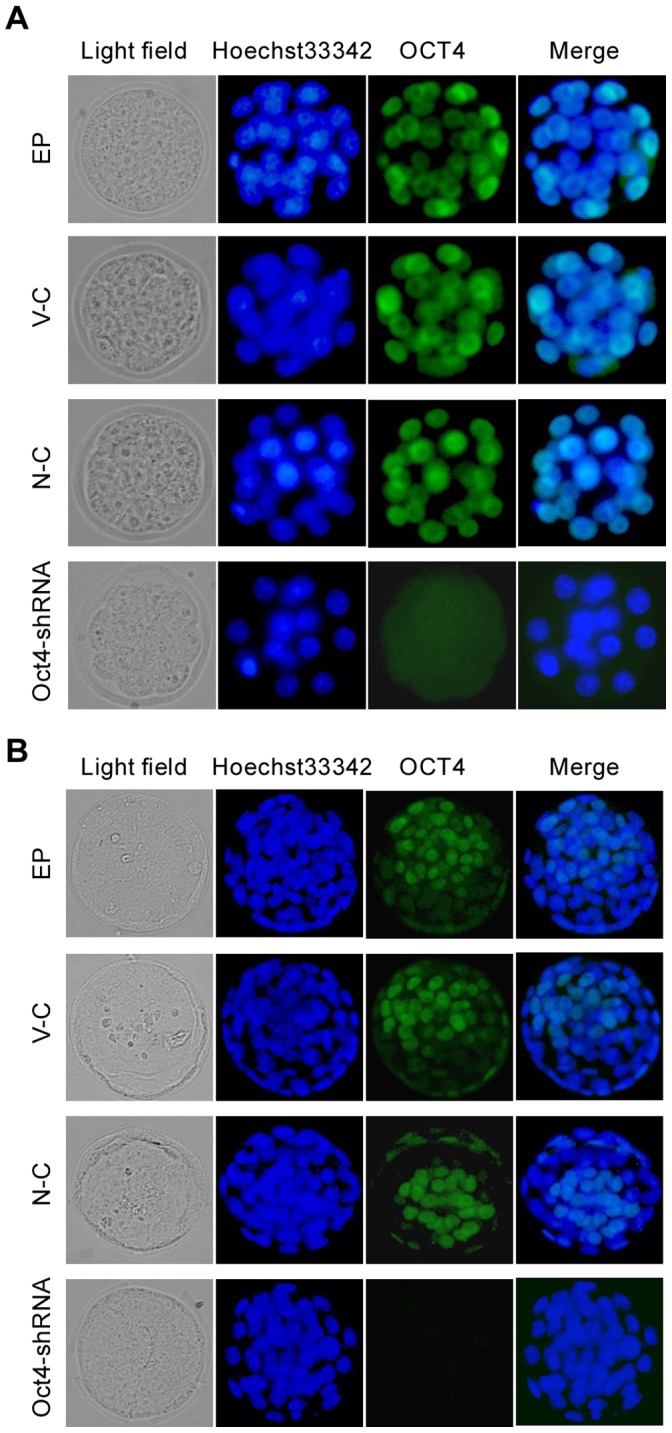
The OCT4 protein level in the multi-cell embryos and blastocysts developed from EP group, vector control (V-C), negative control (N-C) and *Oct4*-shRNA expression vectors electroporation. Nuclear Oct4 expression is absent in *Oct4*-shRNA electroporated embryos at the multi-cell (A) and blastocyst stage (B) but present in embryos obtained from the control groups. Each sample was counterstained with Hoechst 33342 to visualize DNA (blue). The original magnification was ×200.

Parameters such as voltage, pulse duration, number of pulses and repeats for plasmid DNA and morpholinos electroporation were optimized as described above.

**Figure 8:**
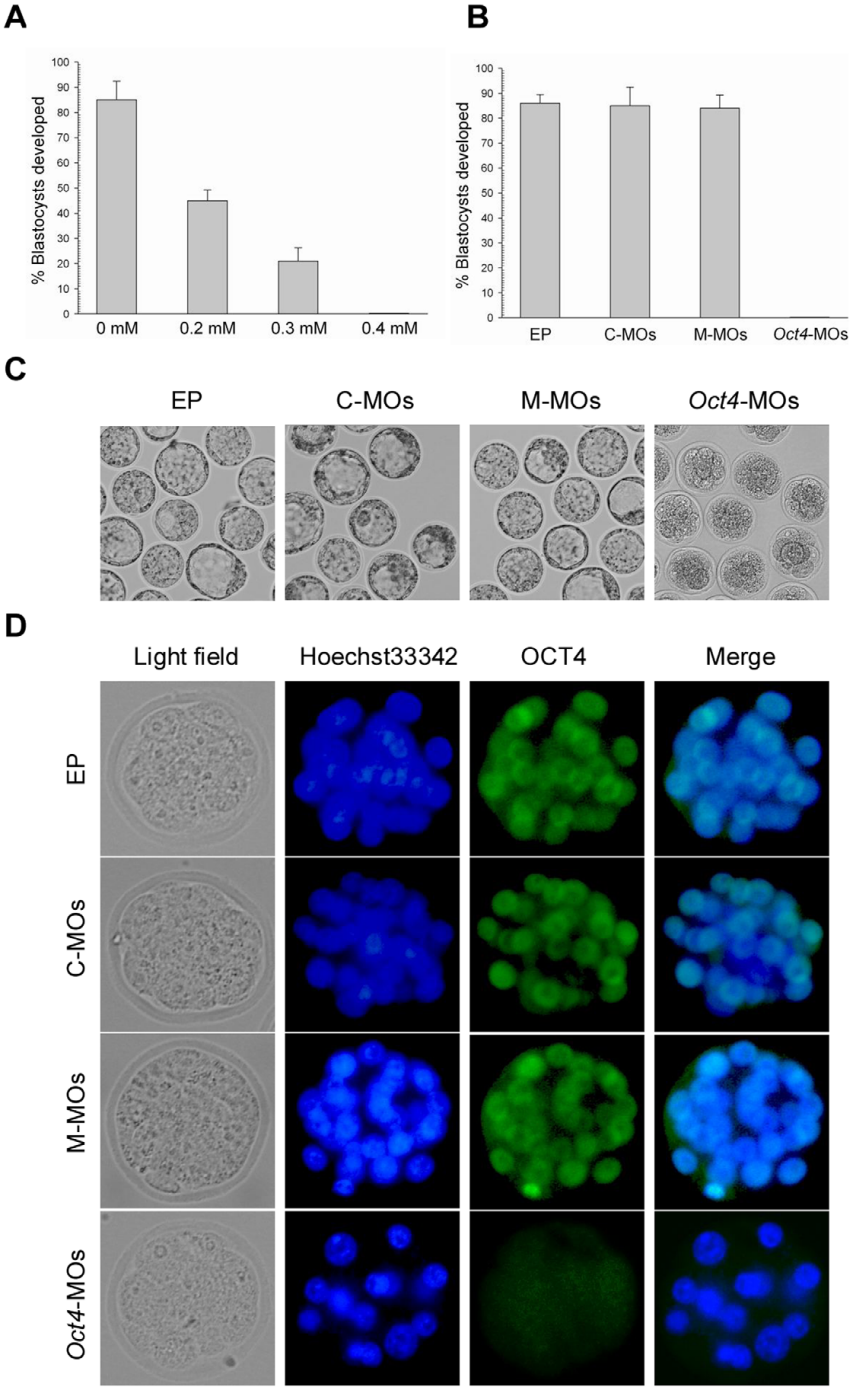
Effects of *Oct4*-targeting morpholinos on early development. (A) Blastocyst development following electroporation of zygotes with customized *Oct4* morpholinos at different concentrations and cultured for 3.5 d. (B) Blastocyst development following electroporation of zygotes with different morpholinos with a concentration of 0.4 mM. (C) Morphological appearance of electroporated zygotes obtained from the control and *Oct4*-MOs groups after being cultured for 3.5 d. The original magnification was ×100. (D) Nuclear Oct4 expression is absent in *Oct4*-MOs electroporated embryos but present in embryos obtained from the control groups. Each sample was counterstained with Hoechst 33342 to visualize DNA (blue). The original magnification was ×200.

### Embryo Electroporation with Plasmid DNA and Morpholinos

A suitably sized drop (30 µl) of the diluted plasmid DNA or morpholinos solution was added between the two electrodes fixed in an electroporation chamber. Zona-weakened embryos were linearly arranged in the flat chamber; simultaneously, the 2001 Electro Cell Manipulator was switched on. A series of electrical square pulses were given to the zona-weakened embryos. After electroporation, embryos were washed three times and cultured in KSOMaa-BSA for 24 h or 3.5 d.

### TUNEL Assays

To evaluate the degree of embryo damage after electroporation, TUNEL (TdT-mediated dUTP nick end labeling) apoptosis assays were carried out with the DeadEnd™ Fluorometric TUNEL System (Promega, Madison, WI, USA) according to the manufacturer’s instructions. Apoptosis assays of blastocysts were performed as described [37].

### Immunofluorescence Staining

After 3.5 d electroporation, embryos were washed three times, fixed in 4% paraformaldehyde for 45 min, permeabilized in PBS/0.2% Triton X-100 for 20 min, blocked in PBS/3% BSA for 2 h at room temperature (RT) and incubated with the primary antibody diluted in PBS containing 1% BSA overnight at 4°C. Anti-CDX2 rabbit polyclonal IgG (sc-134468; Santa Cruz) and anti-OCT4 mouse monoclonal antibody (sc-5279; Santa Cruz) was diluted 1∶50, respectively. After washing in PBS containing 0.3% polyvinylpyrrolidone, embryos were incubated for 1 h at RT with the secondary antibodies of Alexa Fluor 555-labeled goat anti-rabbit IgG (A0452; Beyotime) for CDX2 and Alexa Fluor 488-labeled goat anti-mouse IgG (A0428; Beyotime) for OCT4. The secondary antibodies were diluted 1∶500 with PBS containing 1% BSA. After washing in PBS/0.3% polyvinylpyrrolidone, nuclei were stained with Hoechst 33342 for 5 min. Embryos were observed using a Nikon eclipse Ti-S microscope (Nikon).

### Statistical Analysis

Data were analyzed by one-way ANOVA and LSD tests using SPSS 13.0 software (SPSS Inc., Chicago, IL, USA). The difference was considered statistically significant at P<0.05. Data were presented as mean ± SEM.

## Results

### Treatment of Zona Pellucida with Acid Tyrode’s Solution Improved Electroporation Efficiency

For ease of optimization of treatment times with acid Tyrode’s solution, zygotes were electroporated with modified morpholino oligonucleotides and cultured for 24 h. As shown in Figures 2A and 2B, the level of lissamine fluorescence was progressively enhanced with the increase of treatment times with acid Tyrode’s solution. However, the post electroporation embryo survival rate was gradually decreased (Fig. 2C). Based on an overall analysis of above factors, the optimum treatment time was 20 s for the zona pellucida to be incubated in acid Tyrode’s solution.

### Optimization of Electroporation Parameters

Based on an overall analysis of embryo survival rate and the fluorescence level, parameters such as voltage, pulse duration, number of pulses and repeats for plasmid DNA and morpholinos electroporation were optimized, and are detailed in Table 1. As shown in Figures 3A and 3B, pIRES2-AcGFP1-Nuc and standard control oligonucleotides were all effectively delivered into mouse preimplantation embryos using the optimal parameters.

### Evaluation of Degree of Embryo Damage after Electroporation

To evaluate the degree of embryo damage using TUNEL assay, blastocysts obtained from unelectroporated embryos (C0), EP group, pIRES2-AcGFP1-Nuc and mismatched morpholino oligonucleotides electroporation were performed. As shown in Figure 4, the number of apoptotic cells in blastocysts derived from the four groups was no significantly difference.

### Electroporation did not Affect Cell Differentiation in Blastocysts

To determine whether electroporation would influence normal development of embryos, we investigated the expression levels of OCT4 and CDX2, markers of pluripotency and trophectoderm differentiation [38], [39], respectively. As shown in Figure 5, the final cell fate and differentiation in blastocysts developed from unelectroporated embryos (C0), EP group, pIRES2-AcGFP1-Nuc and mismatched morpholino oligonucleotides electroporation was not affected in terms of the expression of these markers.

### Electroporation of *Oct4*-specific shRNA Expression Plasmids Leads to Down Regulation of its Endogenous Expression

To determine if electroporated plasmid DNA is effective in mouse preimplantation embryos, zygotes were electroporated with customized *Oct4*-specific shRNA expression plasmids. As shown in Figures 6A and 6B, we had determined 100 µg/ml *Oct4*-specific shRNA expression plasmids to be the effective concentration that would allow normal rates of blastocyst development in the control groups. Hence, this concentration was used to subsequent experiments. After electroporation, the embryonic developmental stages and their morphological appearance after being cultured for 3.5 days are presented in Figure 6C. Zygotes obtained from the control groups reached the blastocyst stage. However, the majority of embryos derived from the *Oct4*-shRNA group were arrested the multi-cell stage and had no apparent inner cell mass (ICM). Furthermore, a remarkable reduces of the OCT4 protein level in *Oct4*-shRNA electroporated embryos at the multi-cell stage was observed compared with the control groups (Fig. 7A). Importantly, those minor blastocysts derived from the *Oct4*-shRNA group were not stained by anti-OCT4 antibodies in indirect immunofluorescence experiments (Fig. 7B). Thus, *Oct4*-specific shRNA expression plasmids were able to effectively deliver into mouse preimplantation embryos by electroporation and down regulate endogenous expression of OCT4 protein.

### Reproducible Phenotype of *Oct4*-deficient Embryo by Morpholinos Electroporation

To investigate if electroporated morpholinos are also effective in down regulating genes in mouse preimplantation embryos, zygotes were electroporated with customized *Oct4* morpholinos. As shown in Figures 8A and 8B, 0.4 mM *Oct4* morpholinos was determined to be the effective concentration that would allow normal rates of blastocyst development in the control groups. Hence, this concentration was used to subsequent experiments. Zygotes that were electroporated with the standard control and the mismatched morpholinos or without morpholinos developed into blastocysts with normal morphology after being cultured for 3.5 days. In contrast, zygotes electroporated with customized *Oct4* morpholinos appeared condensed and did not reach the blastocyst stage (Fig. 8C). Most remarkably, OCT4 protein expression was declined sharply in *Oct4* morpholinos electroporated embryos at the multi-cell stage (Fig. 8D). The reproducible phenotype of *Oct4*-deficient embryos, as determined by electroporation, was identical with that previously determined by microinjection [40]. We therefore concluded that electroporation enabled *Oct4* morpholinos to introduce into mouse preimplantation embryos and specifically block the translation of *Oct4* mRNA.

## Discussion

Mouse preimplantation development is characterized by three major transitions and two lineage segregations. The three major transitions are maternal-to-zygotic transition, compaction and blastocyst formation. At the compacting morula stage, trophectoderm and ICM emerge, and later the ICM gives rise to primitive endoderm and epiblast. Each transition or lineage segregation entails pronounced changes in the pattern of gene expression. Thus, research into the function of genes with obvious changes in expression pattern will shed light on the molecular basis underlying preimplantation development. However, conventional approaches to research the gain or loss of gene function are time-consuming, laborious and expensive processes. Here, we described a simplified and effective method–electroporation–of introducing plasmid DNA and morpholinos into mouse preimplantation embryos and verified the effectiveness of this approach.

Before electroporation of preimplantation embryos, the zona pellucida was weakened by the treatment of acid Tyrode’s solution, but not removed. The zona pellucida is important for the embryo’s development; therefore, developmental potential of zona-free mouse embryos is often lower than that of zona-enclosed embryos. We found that the level of lissamine fluorescence was progressively enhanced with the increase of treatment times, suggesting that the thinner the zona pellucida, the more easily a macromolecule can enter under identical electric pulses. However, the thinner the zona pellucida, the lower the embryo viability after electroporation. Thus, the treatment time with acid Tyrode’s solution is very important for electroporation efficiency and embryo survival rate.

We optimized the parameters, such as voltage, pulse duration, number of pulses and repeats, and demonstrated that plasmid DNA and morpholinos were efficiently delivered into mouse preimplantation embryos by electroporation. Compared with the control groups, the number of apoptotic cells in blastocysts developed from electroporated 1-cell embryos was not significantly increased, implying that embryo damage by electroporation might be happening, but to a lesser degree. Furthermore, the expression and localization of OCT4 or CDX2 was not significantly changed in blastocysts developed from electroporated 1-cell embryos, suggesting that this approach does not affect normal development of preimplantation embryos.

To further demonstrate whether electroporated plasmid DNA and morpholinos are effective in mouse preimplantation embryos, we chose *Oct4* as the target gene. *Oct4* is an important regulator of pluripotency that is essential for maintaining ICM cell fate and pluripotency of ES cells [39], [41]. Consistent with previous experiments that knock down *Oct4* expression with siRNA or siRNA vectors via microinjection [10], [42], we confirmed that *Oct4* gene expression was downregulated in preimplantation embryos developed from 1-cell embryos, which were electroporated with *Oct4*-specific shRNA expression plasmids, implying that electroporated plasmid DNA is effective in mouse preimplantation embryos. Furthermore, we also demonstrated that OCT4 protein expression was indeed declined in *Oct4* morpholinos electroporated embryos at the multi-cell stage and the reproducible phenotype of *Oct4*-deficient embryos occurred, as previously reported by microinjection [40], suggesting that electroporated morpholinos are extremely efficient in mouse preimplantation embryos. Thus, electroporated plasmid DNA and morpholinos were able to exert their functions and actually cause expected phenotypes.

Electroporation is a powerful means for spatially and temporally analyzing gene function in development. As far as the gain or loss of gene function is concerned, this approach is easier to implement than classical techniques. This technique has several advantages over those classical techniques for mouse preimplantation development. First, electroporation can save time, economize labor and be more cost-effective. Second, this technique is very simple, and complicated manipulation is not required. Third, the technique is less toxic and highly efficient for embryos. Fourth, morpholinos can be introduced into mouse preimplantation embryos. Finally, this technique can be used to simultaneously co-deliver multiple compounds or plasmids.

Electroporation is a versatile technique applicable to the delivery of a wide range of molecules, including DNA, mRNA, dsRNA, siRNA, morpholinos, peptides, proteins, antibodies, dyes and drugs [31], [43], [44], [45], [46], [47], [48], [49], [50], [51]. Thus, using electroporation in combination with other techniques will extend the knowledge of preimplantation development in the mouse. For example, in combination with microinjection, electroporation can be used to trace blastomere differentiation and two lineage segregations. Moreover, in combination with other molecular techniques, electroporation will be able to provide insights into the developmental stages from zygotes to blastocysts.

In summary, we optimized the parameters such as voltage, pulse duration, number of pulses and repeats, and demonstrated that plasmid DNA and morpholinos were efficiently delivered into mouse preimplantation embryos by electroporation. Meanwhile, the normal development of preimplantation embryos was not affected. Furthermore, we have verified effectiveness of this approach by testing the procedure on the endogenous gene *Oct4*.
